# Exploration of signature based on T cell-related genes in stomach adenocarcinoma by analysis of single cell sequencing data

**DOI:** 10.18632/aging.205687

**Published:** 2024-03-25

**Authors:** Huimei Wang, Nan An, Aiyue Pei, Yongxiao Sun, Shuo Li, Si Chen, Nan Zhang

**Affiliations:** 1 Department of Gastroenterology, The First Hospital of Jilin University, Changchun, China; 2 Department of Gastric Surgery, Sun Yat-sen University Cancer Center, Guangzhou, China; 3 Department of Colorectal and Anal Surgery, General Surgery Center, The First Hospital of Jilin University, Changchun, China

**Keywords:** gastric cancer, single-cell sequencing, immune microenvironment, immunotherapy, prognosis

## Abstract

Background: Gastric cancer (GC) is a leading reason for the death of cancer around the world. The immune microenvironment counts a great deal in immunotherapy of advanced tumors, in which T cells exert an indispensable function.

Methods: Single-cell RNA sequencing data were utilized to characterize the expression profile of T cells, followed by T cell-related genes (TCRGs) to construct signature and measure differences in survival time, enrichment pathways, somatic mutation status, immune status, and immunotherapy between groups.

Results: The complex tumor microenvironment was analyzed by scRNA-seq data of GC patients. We screened for these T cell signature expression genes and the TCRGs-based signature was successfully constructed and relied on the riskscore grouping. In gene set enrichment analysis, it was shown that pro-tumor and suppressive immune pathways were more abundant in the higher risk group. We also found different infiltration of immune cells in two groups, and that the higher risk samples had a poorer response to immunotherapy.

Conclusion: Our study established a prognostic model, in which different groups had different prognosis, immune status, and enriched features. These results have provided additional insights into prognostic evaluation and the development of highly potent immunotherapies in GC.

## INTRODUCTION

Gastric cancer (GC) remains one of the most prevalent malignant tumors, with the fifth highest incidence and mortality rate worldwide [1]. It is assumed that more than one million new cases are diagnosed each year and the 5-year survival rate is only 20% [2, 3]. The most general type is stomach adenocarcinoma (STAD) [4]. While endoscopic treatment or surgery can potentially cure patients in early disease stages, adjuvant therapy is recommended for late-stage patients who have undergone prior surgery and have pathological T3 or T4 lesions or positive lymph node lesions [5, 6]. However, the survival rate of STAD patients in late stages has remained stagnant despite the limited progress made in radiotherapy, chemotherapy, and surgery [4]. For this reason, it is critical to exploit new predictive models to raise the survival rate of STAD patients.

The tumor microenvironment (TME), made up of stromal cells, immunocytes, cytokines, blood vessels, and the extracellular substratum, acts as an important part of the invasion and progress of tumors, significantly affecting the efficacy of immunotherapy [7, 8]. T cells, which recognize and assemble tumor antigen-MHC complexes via T cell receptors, have been shown to be the essential element of antitumor immunity, with chimeric antigen receptor (CAR) T cell therapy having been clinically demonstrated to be an effective immunotherapy [9–11]. Immune checkpoint inhibitors (ICIs) have already been extensively applied, with anti-cytotoxic T-lymphocyte-associated antigen (CTLA)-4 and anti-programmed death-protein (PD)-1 being the most common. In advanced GC patients, it has been shown to be a potentially effective therapeutic measure to target the PD-1/programmed death-ligand 1 (PD-L 1) axis [12, 13]. Nevertheless, just a fraction of STAD patients react to ICIs, and some even experience adverse effects [14, 15]. However, there are relatively few well-rounded structural molecular analyses of T cells in STAD, so we took T-cell-related genes into account to explore further possibilities for gastric cancer treatment by examining the immune landscape.

Single-cell RNA sequencing (scRNA-seq) techniques can potentially supply single-cell genetic analysis data and reveal different immune subpopulations in the TME, creating unprecedented opportunities for immunotherapy and targeted therapies against cancer [16]. Through integration of scRNA-seq and batch RNA-seq data, several studies have successfully developed new prognostic models for cancer [17–19]. Our study used scRNA-seq to synthesize T cell-related genes (TCRGs) data from STAD patients and construct a prospective signature on the basis of these genes, which can serve as a reliable prognostic biomarker, guiding the development of novel chemotherapeutic and immunotherapeutic approaches. We further analyzed the differences in survival status, TME, tumor mutational load (TMB), and drug sensitivity between different risk groups. New approaches to accommodate the heterogeneity and immune landscape in the clinical management of tumors are suggested in our discovery.

## RESULTS

### Analysis of immune microenvironment and establishment of T cell expression profile

**Figure 1:**
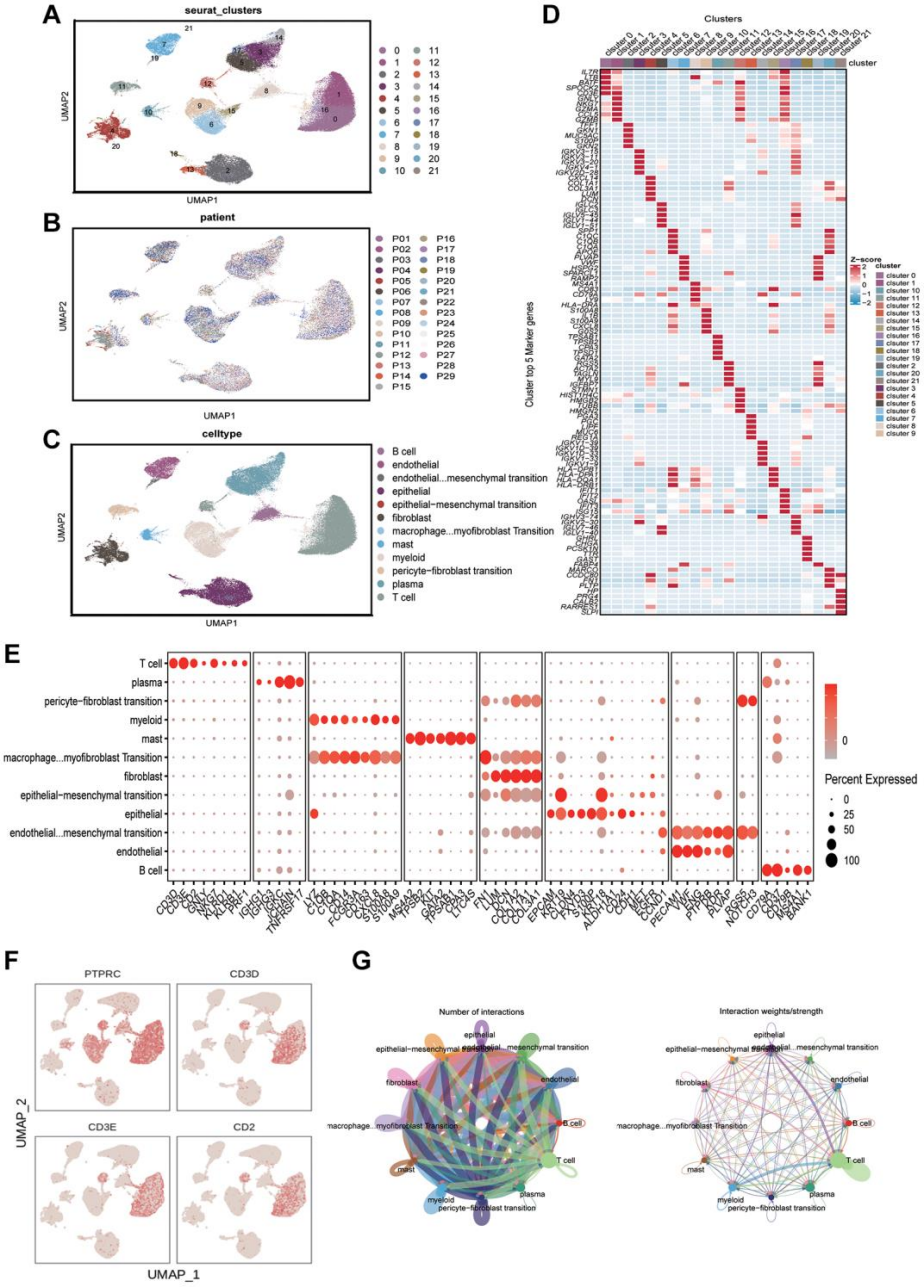
**Analyzing the immune microenvironment.** (**A**) Visualization of 21 subclusters based on UMAP algorithm. (**B**) Display of distribution of cells from different patients after removing batch effects. (**C**) Mark cell subclusters based on Top 5 genes and classic markers. (**D**) A heatmap showing the performance of the top five genes for each subclusters. (**E**) Display of gene expression in different subclusters based on classical cell markers. (**F**) The “featureplot” function describing the allocation of immune cell marker genes and T cell signature genes in the UMAP dimensionality reduction map. (**G**) Based on “cellchat” to display the signal strength of intercellular communication.

**Figure 2:**
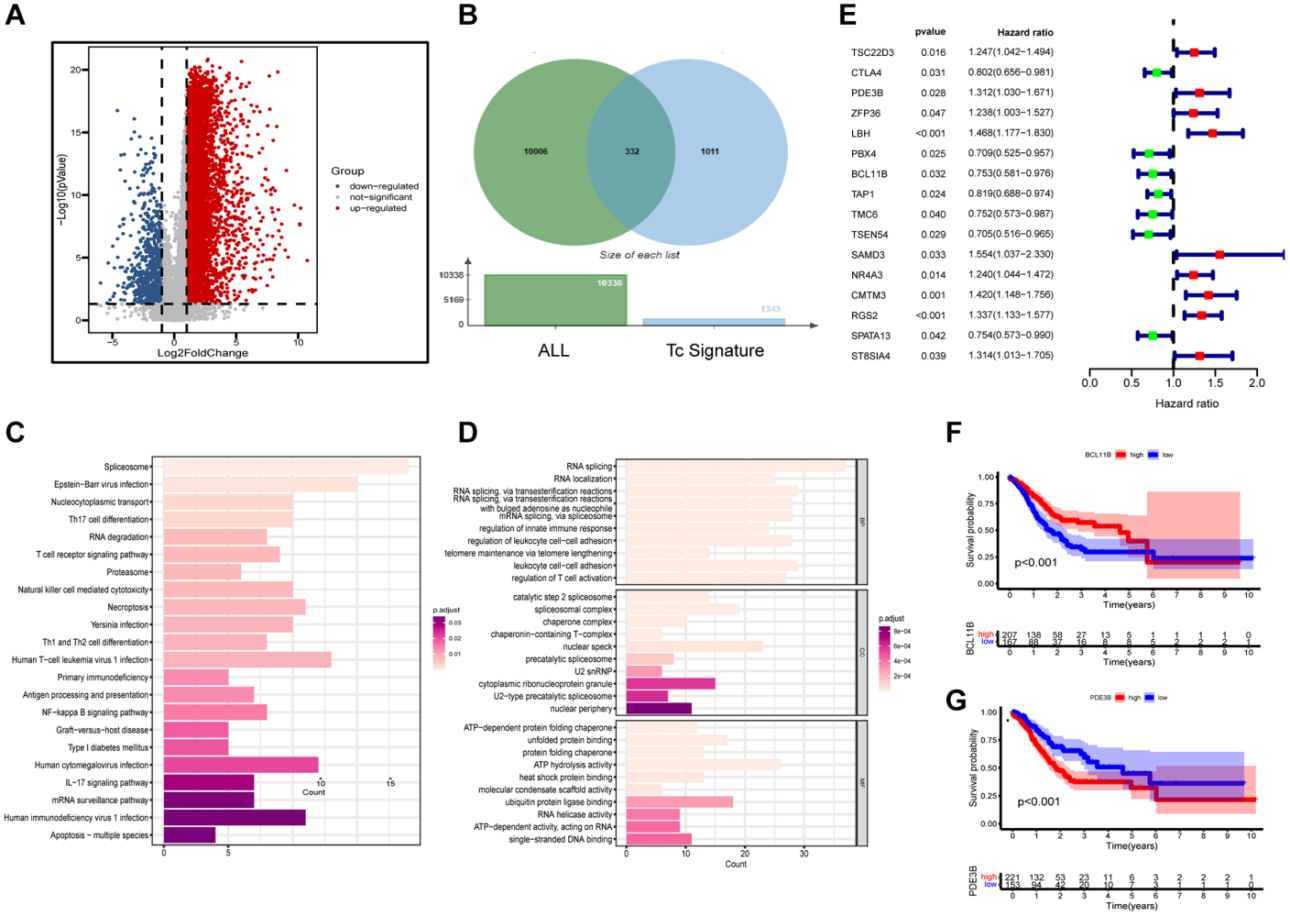
**Functional enrichment and preliminary filters for TCRGs.** (**A**) Volcano map of DEGs in TCGA-STAD cohort. (**B**) Venn diagram, (**C**) KEGG pathway analysis and (**D**) GO analysis of differentially expressed TCRGs. (**E**) Univariate Cox analysis of 16 prognosis-related TCRGs. K-M curves of single prognostic genes, such as (**F**) BCL11B and (**G**) PDE3B.

Data of scRNA-seq for this study were obtained from 29 gastric cancer tissue samples with strict quality control, and 117,916 cells were retained. Meanwhile, we appointed “Harmony” R package to eliminate the batch effect of scRNA-seq data, eliminating batch differences due to different patients (Figure 1B). After log-normalization and dimensionality reduction, a total of 21 single-cell subclusters were obtained (Figure 1A). Then, the “findallmarkers” function was applied to screen the highly expressed genes specific to each subcluster, and displayed the top five genes (Figure 1D). Based on the characteristic genes screened above and the classic markers of cell subclusters, a total of 12 subclusters were identified (Figure 1C, 1E). The “featureplot” function was used to display the expression of classic T cell genes to more accurately define T cell subsets (Figure 1F). Notably, we identified that multiple cell types were in a “transformed” state, such as “pericyte−fibroblast transition”, “epithelial−mesenchymal transition” and so on. In consequential cell communication studies, we found these cells in a transformed state had complex intercellular communication with T cells in the TME (Figure 1G). By reusing the “findallmarkers” function, we identified 1343 signature genes expressed by T cells. We then examined differentially expressed genes (DEGs) from normal tissues and tumors in the TCGA-STAD cohort (Figure 2A). Altogether, 9,574 up- and 764 down-regulated genes were ascertained. Using the Venn diagram, between tumor and normal tissues we screened 332 TCRGs variably expressed (Figure 2B). We carried out Gene Ontology (GO) analysis and Kyoto Encyclopedia of Genes and Genomes (KEGG) pathway enrichment (Supplementary Figure 1) to probe the biological features of the genes. KEGG pathway enrichment showed involvement in apoptosis, mRNA surveillance pathway, and IL-17 signaling pathway (Figure 2C). Through GO analysis, they were found to mainly involve leukocyte cell-cell adhesion, regulation of T cell activation, and nuclear periphery (Figure 2D).

### Construction of the six-gene prognostic signature based on TCRGs

**Figure 3:**
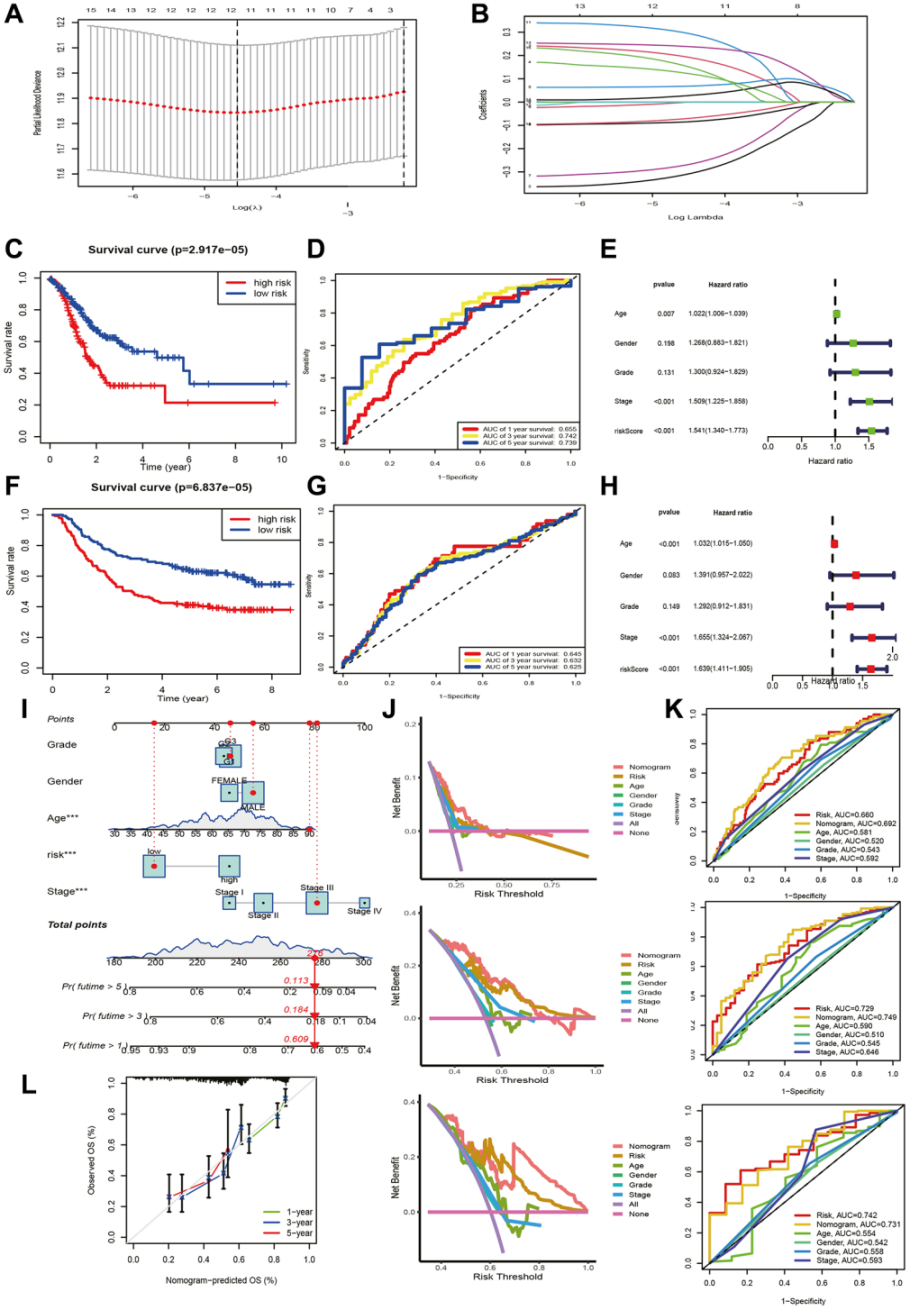
**Prognostic signature and nomogram construction.** (**A**, **B**) LASSO regression analysis. (**C, F**) K-M and (**D**, **G**) ROC curves for training and validation set. Clinical characteristics and riskscore of training set for (**E**) univariate and (**H**) multivariate Cox analyses. (**I**) Construction of nomogram. (**J**) DCA curves for nomogram at 1-year, 3-year, and 5-year. (**K**) ROC curves of nomogram, risk and clinical traits at 1-year, 3-year, and 5-year. (**L**) Calibration curves for nomogram.

Supplementary Table 1 provides patient grouping information for TCGA modeling set and GSE62254 validation set. For constructing a prognostic signature based on TCRGs, TCGA-STAD cohort is our training set. First, univariate Cox analysis was performed on 332 TCRGs, revealing 16 TCRGs significantly associated with prognosis (Supplementary Table 2), with 9 high- and 7 lower risk genes (Figure 2E). The survival curve analysis of these 16 TCRGs indicated that higher risk TCRGs delivery was adversely associated with prognosis (*P* < 0.05) (Figure 2F, 2G; Supplementary Figure 2). Next, 16 TCRGs were submitted to LASSO cox regression analysis based on the optimal lambda value, resulting in the selection of 12 genes for further analysis (CTLA4, PDE3B, ZFP36, LBH, BCL11B, TAP1, TMC6, SAMD3, CMTM3, RGS2, SPATA13, ST8SIA4) (Figure 3A, 3B). At last, six most predictive genes (Supplementary Table 3) were obtained with stepwise multivariate Cox regression analysis. Using correlation coefficients, we developed a model as follows: risk score per patient = (−0.408 × CTLA4 expression) + (0.245 × PDE3B expression) + (0.177 × ZFP36 expression) + (−0.364 × BCL11B expression) + (0.472 × SAMD3 expression) + (0.362 × CMTM3 expression). The STAD patients were grouped into high- and lower risk groups according to neutral values in the training and validation set, which was assessed by ranking high to low risk score.

### Validation and independent predictive effect of the signature

We estimated the prospective meaning of the TCRGs signature. Supplementary Figure 3A, 3B demonstrates the six modeled gene expression levels in two groups, while Supplementary Figure 3C, 3F shows survival status and distribution. The K-M curve showed that the lower risk group also had a significantly longer survival time (*P* < 0.001) (Figure 3C, 3F). Model prediction accuracy was assessed using receiver operating characteristic (ROC) curves yielding the area under the curve (AUC) of 0.655, 0.742, and 0.739 for 1-, 3-, and 5-year survival in the training set (Figure 3D) and 0.645, 0.632, and 0.625 in validation set (Figure 3G). Obviously, a moderately accurate signature based on TCRGs was developed. The clinical characteristics and riskscore of the training group were submitted to univariate and multivariate Cox regression analyses to further explore the independent promotional value of riskscore. We discovered a significant association between riskscore and overall survival (OS) (Figure 3L) as an individual promotional factor (hazard ratio: 1.639, 95% CIs: 1.411–1.905, *P* < 0.001) (Figure 3E, 3H). This finding was confirmed in the validation group (hazard ratio: 1.565, 95% CIs: 1.259–1.944, *P* < 0.001) (Supplementary Figure 3G, 3H).

### Prediction of nomogram

We structured a nomogram to quantify clinical outcomes in STAD patients, which can predict survival by combining riskscore and clinical traits (Figure 3I). The calibration curve indicated a high concordance that exists in the predicted and respected values (Figure 3L). The decision curve analysis (DCA) curve showed the optimal clinical net benefit of the nomogram (Figure 3J), and ROC curve displayed its prediction accuracy (Figure 3K). In summary, the risk-constructed nomogram is more accurate than single factors for predicting prognosis in clinical settings.

### Gene set enrichment analysis (GSEA)

**Figure 4:**
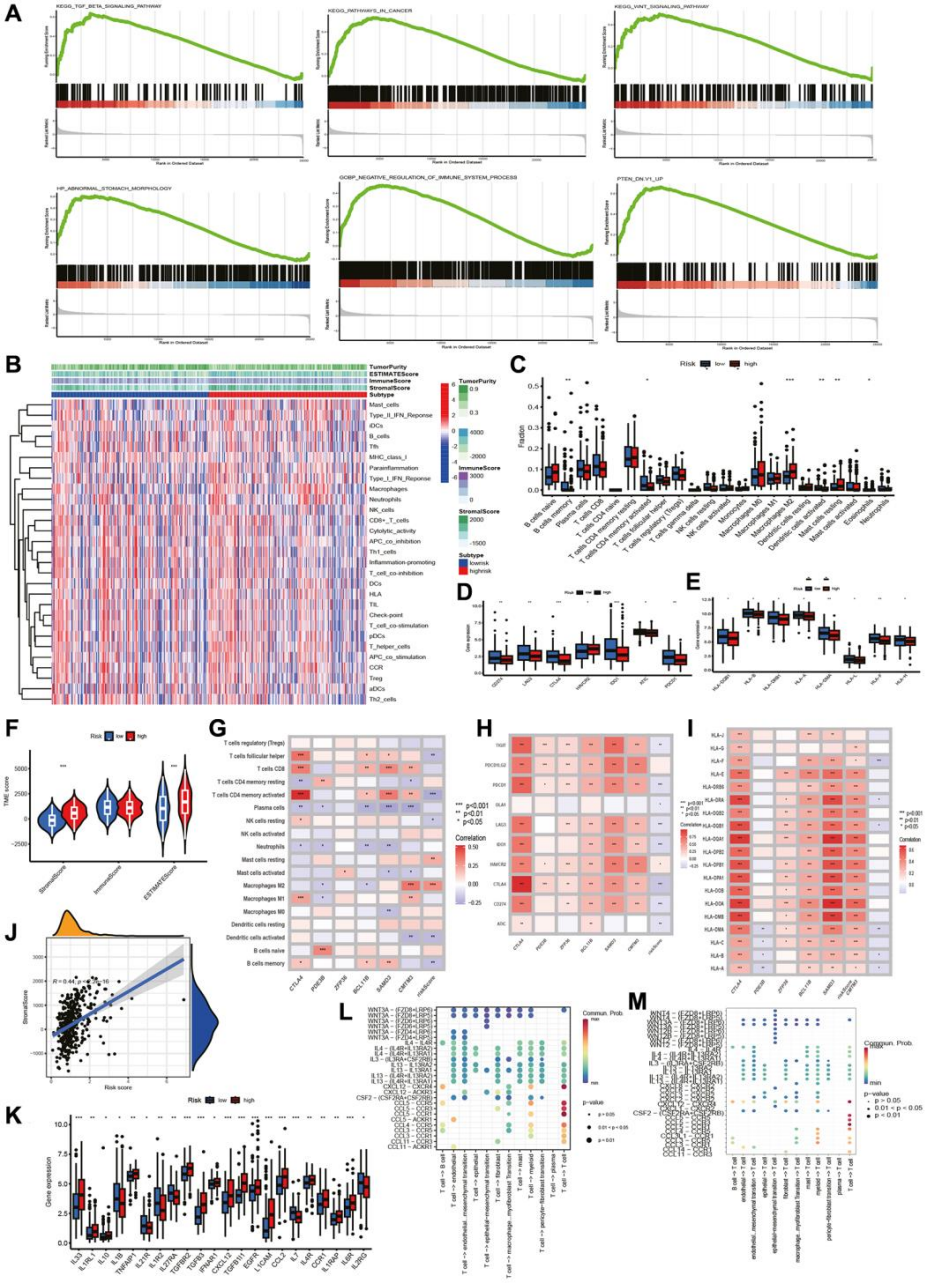
**GSEA and TME analysis. GSEA and TME analysis.** (**A**) Gene set enrichment analysis. (**B**) Heatmap shows risk and immune-related functions relationship. Differential expression of (**C**) 22 immune-associated cells, (**D**) immune checkpoint-associated genes, (**E**) HLA-associated genes, (**F**) stromal, immune and ESTIMATE scores, and (**K**) cytokine-related genes in two groups. Correlation of riskscore with (**G**) immune cells, (**H**) checkpoint-related genes, (**I**) HLA-related genes, (**J**) stromal score. (**L**) T cells initiate cytokine signal exchange between cells. (**M**) T cells receive cytokine signals to terminate intercellular signal communication.

We conducted GSEA to measure functional variations in prognostic markers between two groups (Figure 4A). Six pathways, including the cancer pathways, PTEN, TGF-β and WNT signaling pathway, were more abundant in higher risk group. This observation suggests a greater abundance of pro-oncogenic pathways and immune pathway suppression in higher risk group, leading us to examine of immune related differences between the two groups further.

### Evaluation of TME and immune-related genes

ESTIMATE could assess stromal and immune cell scores. The heatmap revealed differences in TME between two groups (Figure 4B), while the violin plot showed higher stromal score and ESTIMATE score in higher risk group, but no remarkable difference in immune score (Figure 4F). Stromal score and riskscore were found to be positively correlated by correlation analysis, but ImmuneScore was not correlated (Figure 4J). Cibersort profiling of 22 immune cells revealed higher expression of B cells memory, CD4+ T cells memory activated, Dendritic cells activated, and Eosinophils in lower risk group, while macrophage M2 and mast cells in high-risk group (Figure 4C). Comparing the exposure levels of nine immune checkpoints, the expression of all checkpoints was higher in lower risk group except for HAVCR2, while PD-L1 and CD40 was not statistically different (Figure 4D). Human leukocyte antigen (HLA)-related gene expression was also higher in lower risk group (Figure 4E). We then correlated riskscore and immune cells, immune checkpoints, HLA (Figure 4G–4I), and found negative correlations except for positive correlations between riskscore and mast, M2 cells, HAVCR2 immune checkpoints. We concluded that it is possible that lower risk group has superior prognosis following immunotherapy. Furthermore, we compared common cytokine expression levels, to find that IL21R, IL1R2, IL27RA, and IL2RG expressed higher levels in lower risk group (Figure 4K). Cellchat analysis based on scRNA-seq data revealed that the WNT pathway and interleukin chemokine Dun cytokines play significant roles in the complex communication between T cells and other cell types within the GC immune microenvironment (Figure 4L, 4M).

### Gene mutation analysis

**Figure 5:**
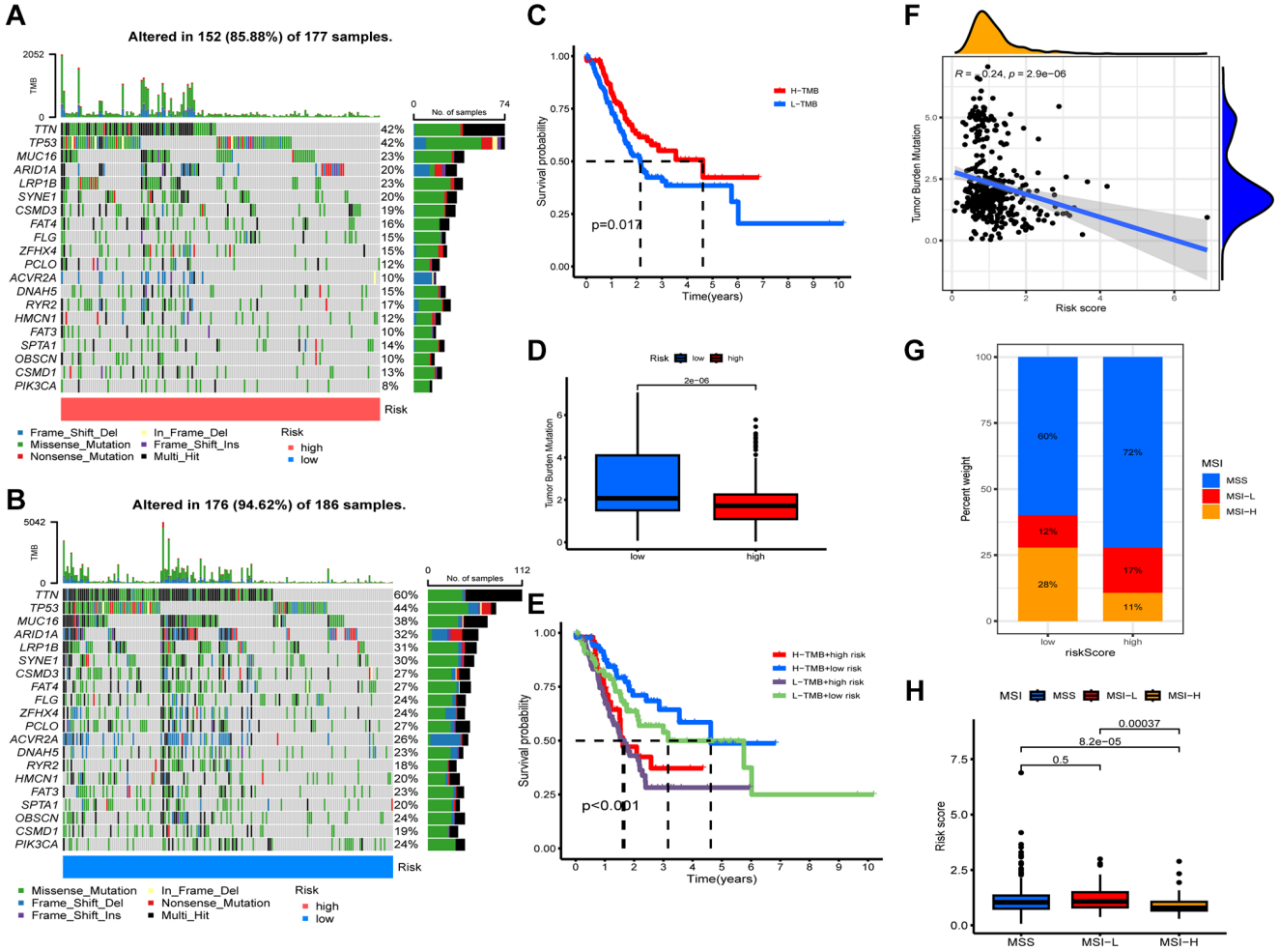
**Characteristics of somatic mutations.** Somatic mutations in different risk groups. (**A**) Higher risk and (**B**) lower risk. (**C**) Survival analysis of low and high tumor mutation burden (TMB). (**D**) Comparison of TMB levels. (**E**) Survival analysis of samples grouped with both risk and TMB. (**F**) Relevance between riskscore and TMB. (**G**, **H**) Relevance between riskscore and MSI.

We analyzed the TMB of STAD patients and identified TTN, TP53, MUC16, ARID1A, and LRP1B as top 5 mutations in both groups (Figure 5A, 5B). Lower risk group had higher TMB (Figure 5D). Figure 5F shows that TMB was negatively associated with riskscore. In addition, patients with higher TMB had a better prognosis, as shown in Figure 5C (*p* = 0.017). We also observed that the best prognosis was observed in the lower risk + higher TMB group, while the worst prognosis was observed in the higher risk + low-TMB group (Figure 5E). Further analysis revealed a significant correlation between low riskscore and MSI-L status, high riskscore and MSS status (Figure 5G, 5H).

### Prediction of immunotherapy response

**Figure 6:**
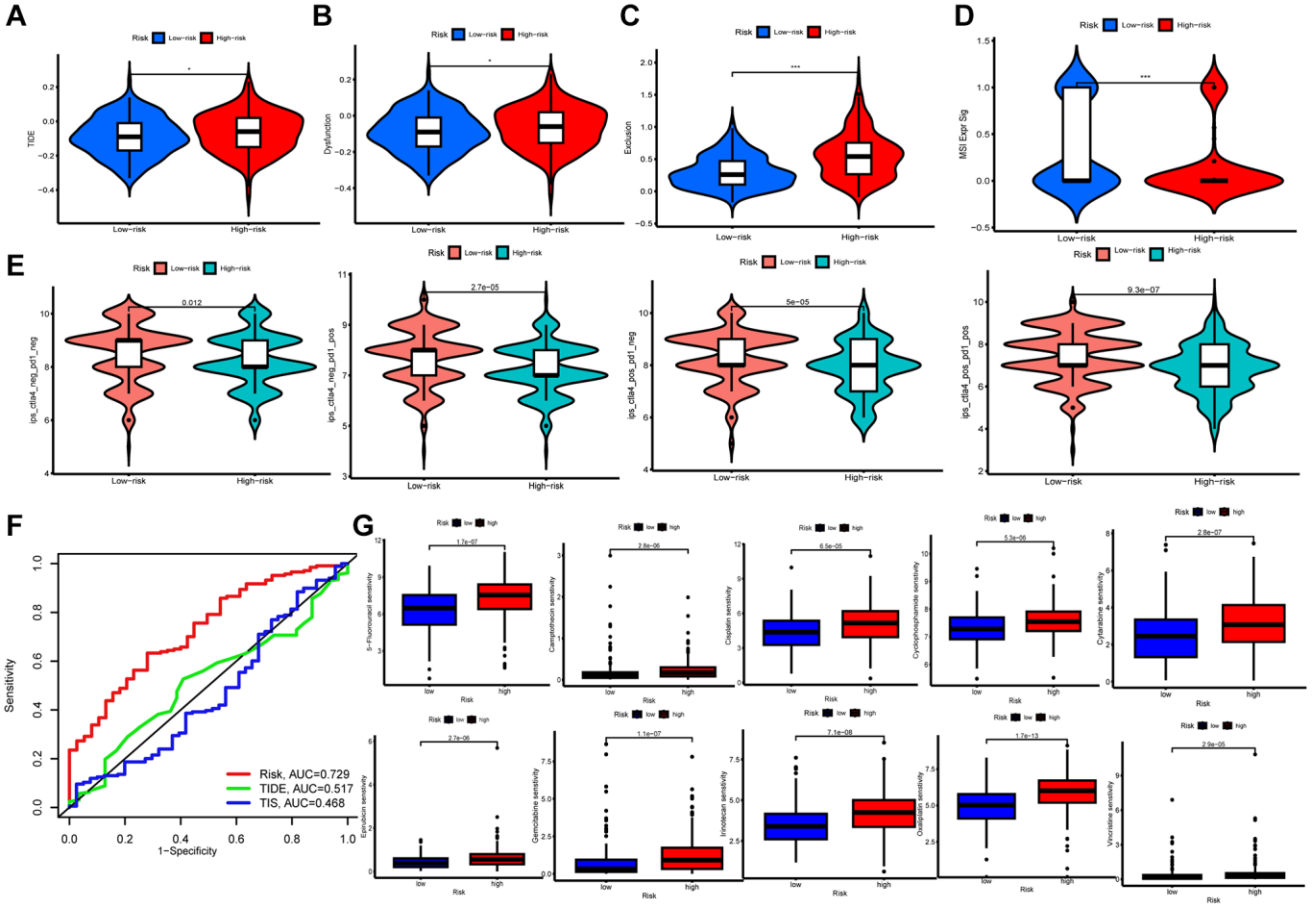
**Immunological evaluation and drug sensitivity analysis.** (**A**–**D**) TIDE, dysfunction, exclusion and MSI Expr Sig scores compared between two groups. (**E**) TCIA for predicting sensitivity to immunotherapy. (**F**) ROC curves comparing risk, TIDE, and TIS at OS. (**G**) Correlation between riskscore and chemotherapeutic sensitivity.

The tumor immune dysfunction and exclusion (TIDE) and The Cancer Immunome Atlas (TCIA) scores were used to assess immunotherapy and immune escape in TCGA-STAD cohort. For all four scores, including TIDE, a poorer response to immunotherapy was surveyed in patients in higher risk group (Figure 6A–6D). TCIA was employed to predict susceptibility to immunotherapy. Outcomes revealed the expression of ips-ctla4-pos-pd1-pos, ips-ctla4-pos-pd1-neg, ips-ctla4-neg-pd1-neg, and ips-ctla4-neg-pd1-pos were higher in lower risk patients (Figure 6E). It indicated that the lower risk samples had better efficacy, regardless of CTLA 4 and PD-1 status. Then, we compared the accuracy of risk, TIDE score, and immune tumor infiltrating lymphocytes (TIL) score in predicting prognosis, and the TCRGs signature had the highest predictive accuracy (Figure 6F).

### Drug susceptibility analysis

To ascertain the medical meaning of this signature, we also sought to determine the 50% maximum inhibitory concentration (IC_50_) levels of chemotherapeutic drugs (Figure 6G). Lower risk patients were found to have a lower IC_50_ for both chemotherapy and targeted medications such as 5-Fluorouracil, Camptothecin, Cisplatin, Cyclophosphamide, Cytarabine, Epirubicin, Gemcitabine, Irinotecan, Oxaliplatin, and Vincristine. Therefore, the developed model could serve as a predictor for selecting anti-cancer drugs.

## DISCUSSION

Accelerated advancement of scRNA-seq technology has resulted in an increasing number of studies exploring the structural characterization of TME [20, 21]. Immune cell regulation, particularly T cell activation and suppression, is strikingly shaping the control of diverse immune responses within TME [22, 23]. T cells occupy an important part in TME and are involved in immune escape and tumor progression [24]. Cancer immunotherapy using ICIs has become an effective therapy for tumor treatment; however, one of the reasons for its poor efficacy is the presence of immunosuppressive mechanisms in TME that diminish the effector function of CD8 tumor-infiltrating lymphocytes (TILs) [25], and T-cell up-regulation of PD-1 has become a major marker of T-cell dysfunction [26]. This study was designed to bioinformatically analyze the sc-RNA-seq spectrum of gastric cancer and identify TCRGs in gastric cancer tumor tissues. We resolved the gastric TME, analyzed multiple cell subtypes in the transformed state, anatomized the interaction of T cells with other cells in the microenvironment, and identified T cell-specific gene expression. We then mined the TCGA database and developed a prognostic signature of six genes with fully validated predictive power in the GEO cohort. Subsequently, we noticed immune cell infiltration, the expressions of immune checkpoints and HLA were more abundant in the lower risk samples, and the response to immunotherapy was notably outperformed. In this research, a new prognostic model for gastric cancer was established based on T-cell-related genes in TME, which provides a theoretical basis for judging the efficacy of immunotherapy and developing new immunotherapy targets.

The prognostic signature of this study consisted of six TCRGs, including CTLA4, PDE3B, ZFP36, BCL11B, SAMD3, and CMTM3. CTLA-4 is a crucial adverse regulator in T cell responses, operating to maintain the tolerance of T cells to a self-antigen [27]. CTLA-4 limits the initiation of nascent T cells of lymphoid through B7 interactions, and Anti-CTLA-4 monoclonal antibodies (mAbs) exert immunotherapeutic effects mainly by depleting regulatory T (Treg) cells in TME and blocking transendocytosis of B7 in dendritic cells (DCs) [28, 29]. PDE3B functions importantly in regulating energy metabolism, particularly regulating compartmentalized cyclic adenosine monophosphate (cAMP)-signaling pathways [30], and its downstream enzyme, which breaks down triglycerides into fatty acids and glycerol, is associated with cAMP levels and lipolytic activity [31]. ZFP36 autonomously regulates the early activation kinetics of T cell by inhibiting the expression of activation markers, limiting T cell expansion and promoting apoptosis to inhibit enrichment and transformation of mRNA targets [32, 33]. CL11B binds directly to Id2 and represses it, a gene that encodes an E protein antagonist, to facilitate effector CD8 T cell expansion, memory formation and cytotoxic functions, and to promote and regulate NK cells differentiation [34]. SAMD3 engages in cell cycle control and also has the ability to bind to RNA and lipids, contributing to memory differentiation in CD8 T cells and is also highly expressed in NK cells [35]. CMTM3 is a neoplasm inhibitory gene that inhibits the tumorigenicity of GC cells mediated by epidermal growth factor receptor (EGFR) by increasing the activity of Rab5. CMTM3 knockdown promotes GC metastasis through the STAT3/Twist1/EMT signaling pathway [36]. The TCRGs model established in this study provides a possible molecular mechanism to further study the clinical aspects of STAD.

Further validation of the prognostic model performance built on six TCRGs was tested in the GEO cohort. Consistent results were observed across two lineups, indicating that the signal had good robustness and repeatability. We also setup a nomogram, and used AUC curves, calibration curves, and DCA curves to visualize and test the accuracy of the predictions. Consequently, the nomogram can direct the development of personalized predictive models of STAD patients. Performing GSEA analysis on signature genes identified immune and tumor pathways can be enrolled markedly. Following this, we aimed to reveal the differential immune infiltration patterns screened by immune-related features. The relationship between riskscore and TME was investigated. First, higher risk group had higher stromal score and ESTIMATE score. Subsequently, 22 levels of immune cell infiltration demonstrated a greater proportion of activated memory CD4+ T cells, memory B cells, activated Dendritic cells, and Eosinophils in lower risk patients, indicating that a relatively active antitumor immune response may be present [37]. It was noted that higher risk group had more resting Macrophages M2 and Mast cells, and high macrophage infiltration rates can lead to poor prognosis in gastric cancer [38], and it has been shown that macrophages can significantly promote gastric cancer metastasis by enhancing the EMT process [39]. Several common immune checkpoints (CD274, CTLA-4, LAG3, PD-CD1, ATIC) were identified to be highly expressed in low-risk populations for better clinical application of ICI. HLA acts as an antigen presenting factor regulating immune response in gastric cancer [40]. Lower risk group had higher expression of HLA-related genes, indicating a more active local immune response. We conclude that lower risk group had greater likelihood of benefiting from immunotherapy. Analysis of TMB revealed a higher tolerance in lower risk group and a meaningful association between low riskscore and MSI-L status, which is consistent with our previously obtained findings. Finally, to study sensitivity to chemotherapeutic agents for clinical application, we performed pharmacosensitivity analysis in different risk groups and detected that 5-Fluorouracil, Camptothecin, Cisplatin, Cyclophosphamide, Cytarabine, Epirubicin, Gemcitabine, Irinotecan, Oxaliplatin and Vincristine had lower IC_50_.

Although this study developed a novel and validated prognostic model, it still exists a few limitations. First, our study is a retrospective survey conducted on public database and should be prospectively and multi-center verified, in addition to validation or research with our own sequencing data. We identified six genes that were highly correlated with prognosis, and explored the biological significance, and we will further analyze their downstream mechanisms by means of immunohistochemistry and animal experiments in the future to explore how TCRGs affect TME and thus prognosis. In addition, T-cells include exhausted T-cells, tissue-resident T-cells and other subtypes, and we plan to further subdivide the cell subtypes and explore the expression of these gene markers in them and the biological functions they play, so as to further explore the potentials of these gene markers, as well as the signature, in immunotherapy, in order to develop more targeted therapeutic agents for gastric cancer.

Collectively, we combined RNA-seq data to develop a prognostic signature based on TCRGs as well as discussing the differences of TME, TMB, and drug sensitivity in two groups, thus providing novel perspectives for further treatment of STAD clinically.

## MATERIALS AND METHODS

### Data collection

Data were collected from various databases. The single-cell sequencing data for 29 primary gastric adenocarcinoma samples were derived from the GEO database (GSE183904). Clinical dataset and genomic details of STAD patients were derived from 379 tumor samples and 34 normal samples in The Cancer Genome Atlas database (TCGA). After removing tumor samples without survival information, 367 tumor samples were used. Additionally, GSE62254 (*n* = 300) was downloaded in the GEO database to verify the predictive capability of the signature. We corrected for abiotic bias with “ComBat” algorithm of “sva” package between different datasets [41].

### Characterizing T-cell marker genes with scRNA-seq data

We analyzed scRNA-seq data with “Seurat” R package. Initially, we used the “Seurat” R package to read 10 × Genomics single cell sequencing data and create Seurat objects. We did quality check by removing cells expressing fewer than 100 genes or more than 5% mitochondrial genes. Only genes that are expressed in a minimum of three single cells were preserved. Then, we used the function "NormalizeData" to normalize the scRNA-seq results and the function “FindVariableFeatures” to identify the upper 2000 high variant genes. Next, perform principal component analysis (PCA) by applying "RunPCA" function, and the first 30 dimensions were selected to decrease the dimensionality of scRNA-seq data for the upper 2000 genes. Then we removed batch effects for 21 samples using the “RunHarmony” function. We clustered single cells into different subgroups by “FindNeighbors” and “FindClusters” functions (dim = 50 and resolution = 0.5). We performed uniform manifold approximation and projection (UMAP) to visualize the clustering units at the two-dimensional level. After annotating by classical cell subpopulation marker genes, DEGs were counted for each cluster with the Wilcoxon-Mann-Whitney test employing the “FindAllMarkers” function in the “Seurat” package. Cut-off thresholds were adjusted for *p*-values < 0.01 and |Log2(foldchange)| > 1 to determine the maker genes.

### Cell communication analysis

The “cellchat” function was applied to analyze the complex intercellular communication network in the TME. We used the major signal inputs as well as the major signal outputs of cell subpopulations in the TME from its built-in database, and the “netVisual_circle” function to visualize the strength of the intercellular communication network between the target cell cluster and other cell clusters throughout the TME.

### TCGA data analysis and functional enrichment

DEGs were accessed with “limma” package [42]. The threshold used was FDR < 0.05 and |Log2(foldchange)| > 1. Venn diagrams were then used to identify TCRGs with differential expressions. For GO analysis and KEGG pathway analysis [43, 44], we employed “ClusterProfiler”, “org.Hs.eg.db”, “GOplot”, and “enrichplot” packages.

### Establishment and confirmation of prognosis signature

Initially, the differential expressions of TCRGs were analyzed by single factor Cox regression (*p* < 0.05) and we drew Kaplan-Meier curves for each gene, which was gained from the "survival" and “survminer” packages. LASSO Cox regression using the “glmnet” package (*p* < 0.05) was done to prevent overfitting. Optimal λ was chosen to eliminate similar genes. Next, a signature of TCRGs was developed by stepwise multivariate Cox regression analysis, resulting in riskscore for each patient = βgene1 × Expgene1 + βgene2 × Expgene2 + ⋯ + βgenen × Expgenen. Patients were divided into high-risk and low-risk groups in terms of median riskscore. We performed survival analysis with the Kaplan-Meier method, comparing the diversity of OS between the two groups using the long rank test. For drawing ROC curves and computing the AUC, we used the “survivalminer” and “survivalROC” packages. The TCRGs signature was verified in 300 STAD cases in GSE62254, and riskscore was derived for every sample considering the identical equation. Again, to affirm the predictive power of the feature, survival analysis and ROC curves were plotted.

### Establishment and verification of nomogram

Utilizing clinical features and TCRGs signature, a nomogram was created with "rms" package to estimate the 1-, 3-, and 5-year survival rate. A score for each variable was assigned by the nomogram scoring system and they were combined to obtain the total score for a single sample [45]. The signature’s precision was measured by calibration curves as well as ROC curves, while net clinical benefit was assessed using DCA curves [46].

### Gene set enrichment analysis

Functional and pathway variations between risk groups were studied by GSEA [47]. GSEA software (http://software.broadinstitute.org/gsea) was used for loading GO and KEGG gene sets, as well as phenotype tag expression files. The threshold was *P* < 0.05 and FDR < 0.25 to make significance.

### Assessment of tumor immune microenvironment

Evaluation of stromal and immune cells within the tumor tissue was performed with “estimation” package to determine immune score, interstitial score, estimated score and tumor purity in STAD patients [48]. Heatmap was generated utilizing the “pheatmap” package. The CIBERSORT algorithm [49] was applied in order to acquire the infiltrative characteristics in 22 types of immune cells, while we employed the single sample GSEA (ssGSEA) to determine the activity of immune cells and immune functions in each sample. The expression of immune checkpoints and HLA-related genes were compared between two groups with Wilcoxon test, and correlations between immune cells, immune checkpoints, HLA-related expression and risk scores were analyzed.

### Analysis of TMB

TMB is meant to be the count of somatic mutations, insertions and deletions in the coding regions of the genome per million bases. We obtained patient somatic cell mutation data in TCGA-STAD cohort. TMB was computed for each sample, and “maftools” package was deployed for the visualization of somatic mutation patterns [50]. K-M curves were applied to assess the diversity of OS between different risk groups. The association between TMB and riskscore was also evaluated.

### Immune status in risk groups and drug sensitivity

The TIDE website (http://tide.dfci.harvard.edu) features the TIDE score files, which were assessed immunotherapy and immune escape in STAD patients [51]. We analyzed the immune genome and obtained the immune treatment scoring file with the TCIA online platform (https://tcia.at/home) [52]. The variation between different groups for these scores was then analyzed. We also used the “pRRophetic” software package [53] to calculate chemotherapy response with IC_50_.

### Statistical analysis

Statistical analyses were performed by R software version 4.2.0 (http://www.R-project.org). The levels of significance had the following annotations: ^*^P < 0.05, ^**^P < 0.01, and ^***^P < 0.001.

### Data availability

Publicly available datasets were analyzed in this study. These data can be found here: https://portal.gdc.cancer.gov/ and GEO (GSE62254) database.

## Supplementary Materials

Supplementary Figures

Supplementary Tables
