# Supplementary material for: Exploration of signature based on T cell-related genes in stomach adenocarcinoma by analysis of single cell sequencing data
Source: Aging (Albany NY). 2024 Mar 25;16(7):6035–53. doi: 10.18632/aging.205687 (PMC11042963; doi:10.18632/aging.205687)
Supplement: Supplementary Figures [file aging-16-205687-s001.pdf]

SUPPLEMENTARY FIGURES

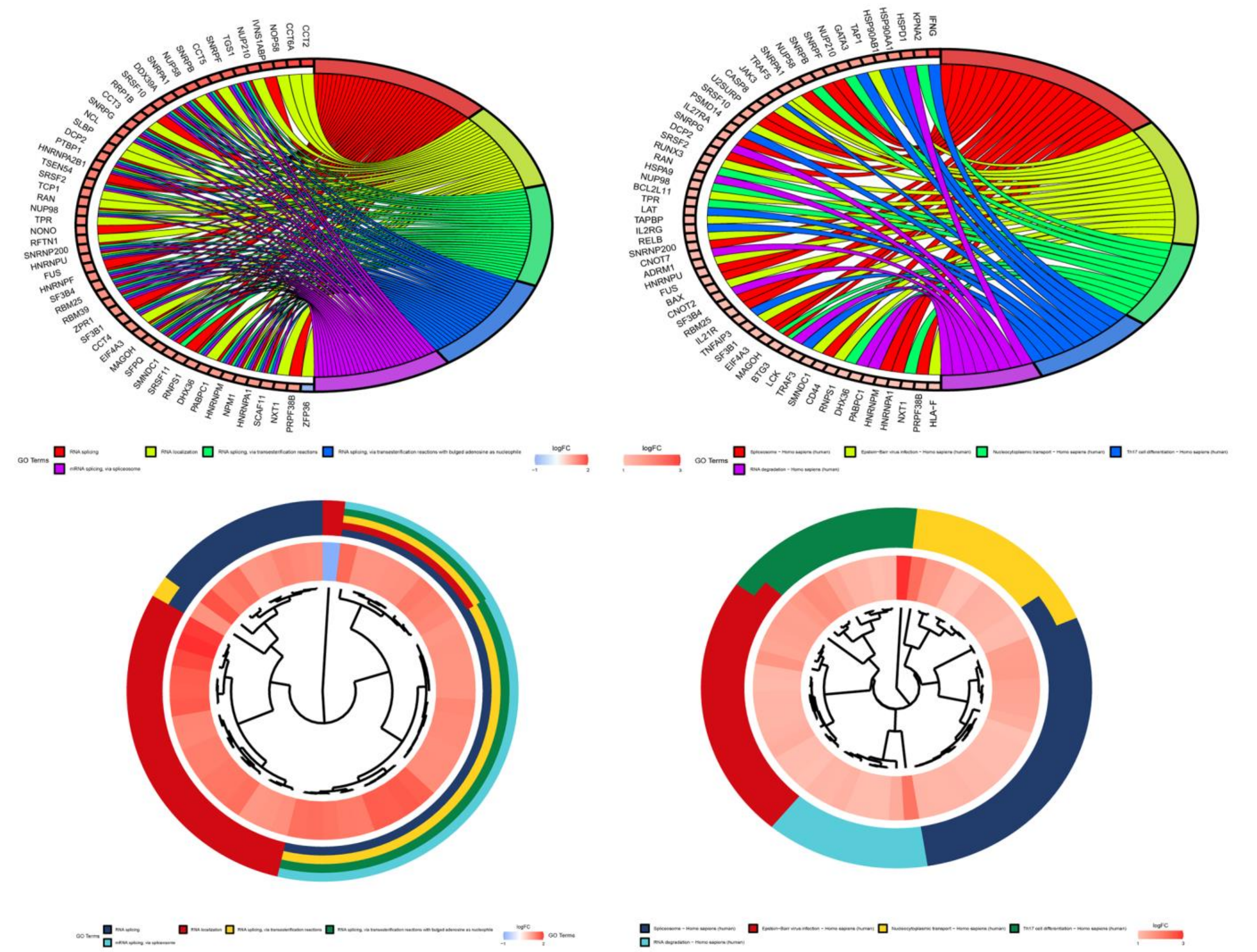

Supplementary Figure 1. Functional enrichment for differentially expressed TCRGs. Concentric circle diagram of KEGG pathway analysis and GO analysis.

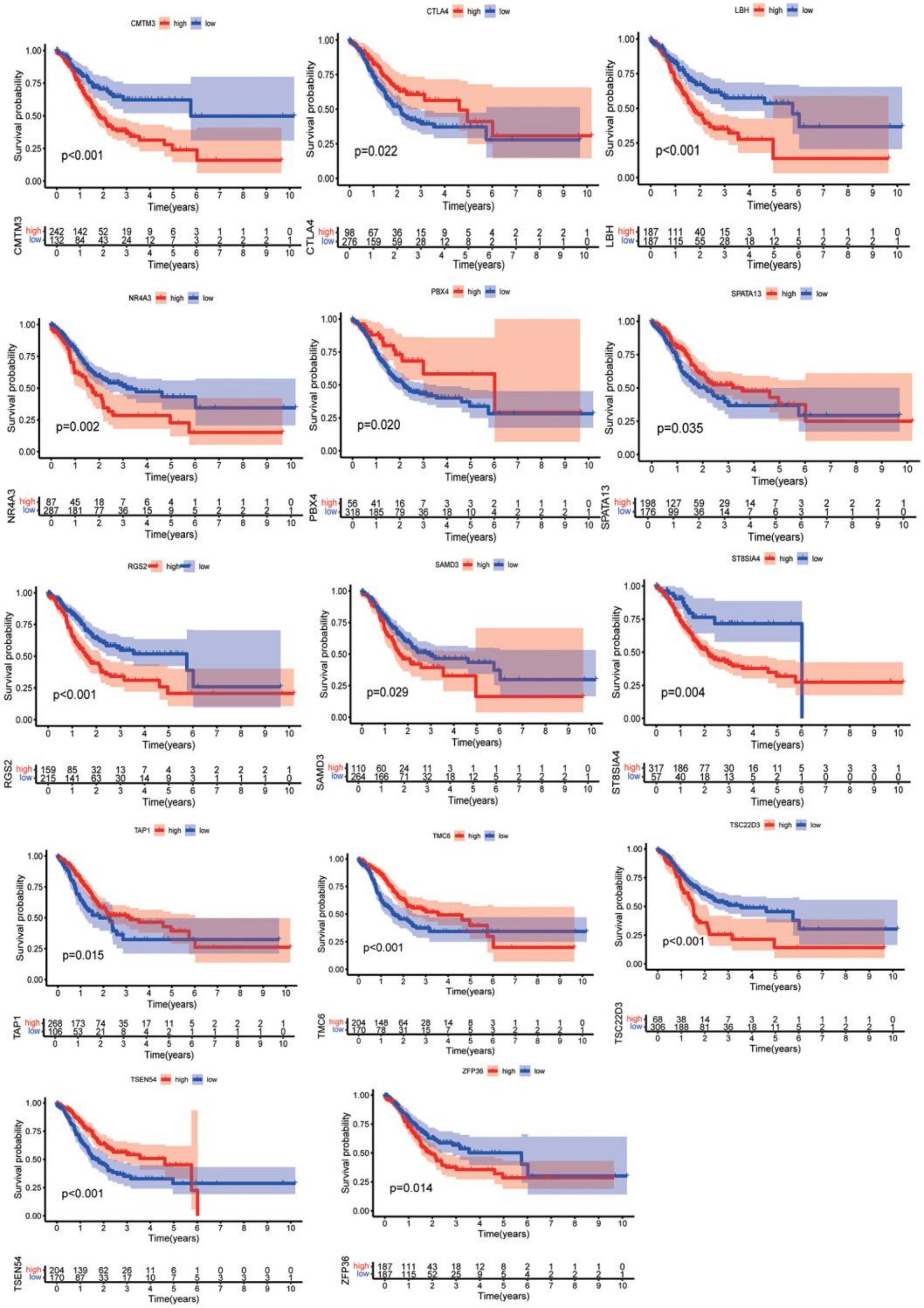

Supplementary Figure 2. K-M curves of single prognostic genes.

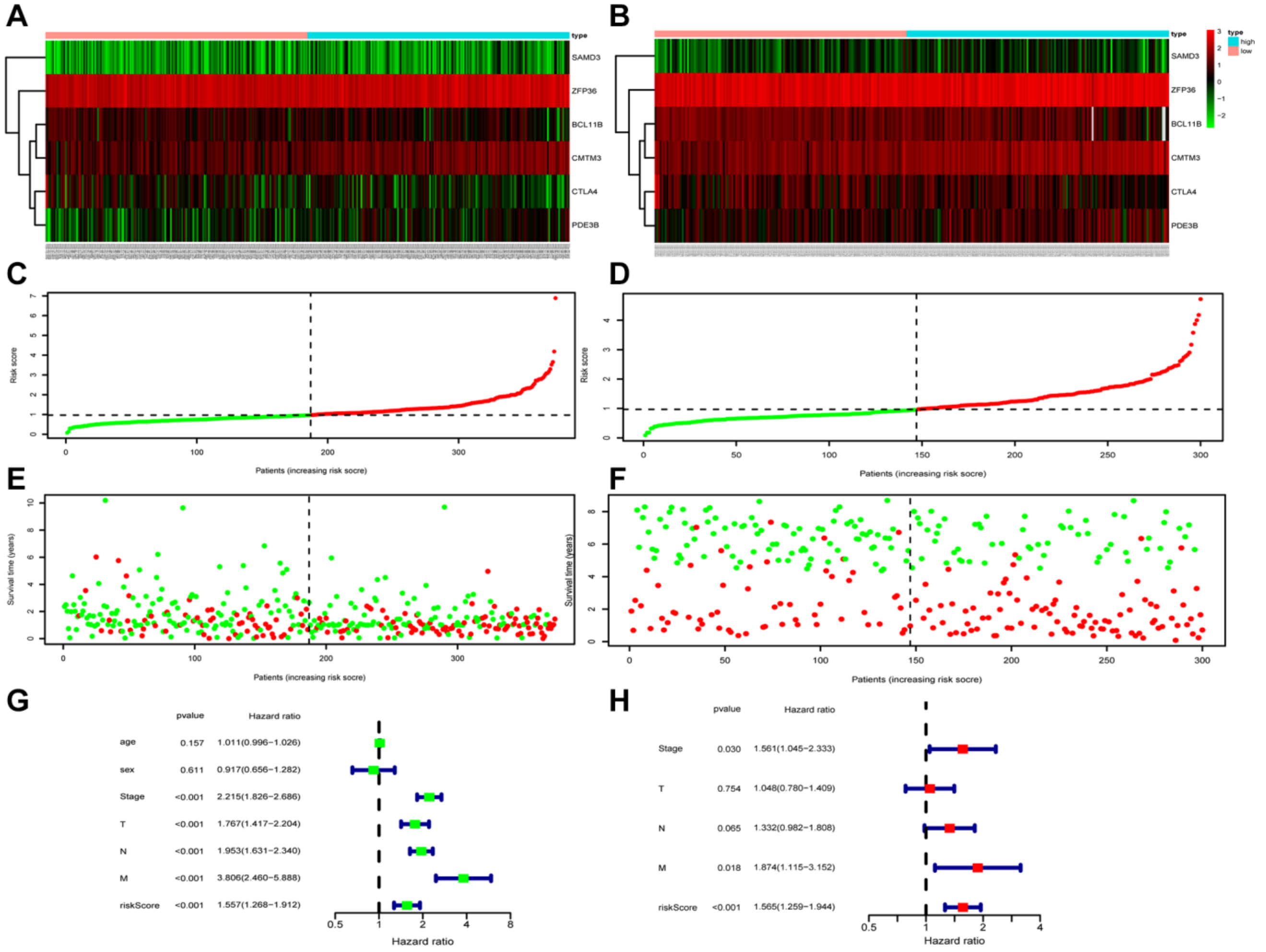

Supplementary Figure 3. Survival status in different groups and independent prognostic analysis in GSE62254. Heatmap for the expression characteristics of 6 modeled genes in (**A**) training set and (**B**) validation set. Distribution of riskscore in (**C**) training set and (**D**) validation set. Distribution of each patient's OS in (**E**) training set and (**F**) validation set. (**G**) Univariate and (**H**) multivariate Cox analysis of clinical characteristics and risk in validation set.
