# Supplementary material for: Exploration of signature based on T cell-related genes in stomach adenocarcinoma by analysis of single cell sequencing data
Source: Aging (Albany NY). 2024 Mar 25;16(7):6035–53. doi: 10.18632/aging.205687 (PMC11042963; doi:10.18632/aging.205687)
Supplement: Supplementary Tables [file aging-16-205687-s002.pdf]

SUPPLEMENTARY TABLES

Supplementary Table 1. Clinical subgroup statistics in TCGA-STAD cohort and GSE62254 cohort.

Covariates	Type	Total	Test	Train
**Age**	≤65	340 (50.45%)	172 (57.33%)	168 (44.92%)
	>65	333 (49.41%)	128 (42.67%)	205 (54.81%)
	Unknown	1 (0.15%)	0 (0%)	1 (0.27%)
**Gender**	Female	238 (35.31%)	101 (33.67%)	137 (36.63%)
	Male	436 (64.69%)	199 (66.33%)	237 (63.37%)
**Stage**	Stage I	82 (12.17%)	30 (10%)	52 (13.9%)
	Stage II	210 (31.16%)	97 (32.33%)	113 (30.21%)
	Stage III	248 (36.8%)	96 (32%)	152 (40.64%)
	Stage IV	111 (16.47%)	77 (25.67%)	34 (9.09%)
	Unknow	23 (3.41%)	0 (0%)	23 (6.15%)
**T**	T1	21 (3.12%)	0 (0%)	21 (5.61%)
	T2	266 (39.47%)	186 (62%)	80 (21.39%)
	T3	259 (38.43%)	91 (30.33%)	168 (44.92%)
	T4	118 (17.51%)	21 (7%)	97 (25.94%)
	Unknown	10 (1.48%)	2 (0.67%)	8 (2.14%)
**M**	M0	609 (90.36%)	273 (91%)	336 (89.84%)
	M1	50 (7.42%)	27 (9%)	23 (6.15%)
	Unknown	15 (2.23%)	0 (0%)	15 (4.01%)
**N**	N0	150 (22.26%)	38 (12.67%)	112 (29.95%)
	N1	231 (34.27%)	131 (43.67%)	100 (26.74%)
	N2	153 (22.7%)	80 (26.67%)	73 (19.52%)
	N3	123 (18.25%)	51 (17%)	72 (19.25%)
	Unknown	17 (2.52%)	0 (0%)	17 (4.55%)

Supplementary Table 2. Univariate Cox analysis of differentially expressed TCRGs.

ID	HR	HR.95L	HR.95H	*p*-value
**TSC22D3**	1.247390192	1.04160897	1.49382574	0.016254411
**CTLA4**	0.802257011	0.656394473	0.980532802	0.031398933
**PDE3B**	1.312037635	1.030111424	1.671122866	0.027784017
**ZFP36**	1.237549084	1.003282025	1.526517667	0.046528572
**LBH**	1.46803763	1.177382365	1.830445697	0.000648292
**PBX4**	0.709013423	0.525328668	0.956924806	0.02459099
**BCL11B**	0.753106303	0.580945207	0.976286743	0.032258763
**TAP1**	0.818587069	0.688308638	0.973523725	0.023613337
**TMC6**	0.751788032	0.572525375	0.987179382	0.040091219
**TSEN54**	0.705467152	0.515530036	0.965382943	0.029249701
**SAMD3**	1.554138556	1.03674294	2.329744969	0.032788817
**NR4A3**	1.239645941	1.043627503	1.472481372	0.014436362
**CMTM3**	1.419781294	1.148138597	1.755693022	0.001216714
**RGS2**	1.337070239	1.133311226	1.577463262	0.000574322
**SPATA13**	0.753666261	0.573484162	0.990459492	0.042485568
**ST8SIA4**	1.314191148	1.013241283	1.704528233	0.039484618

Supplementary Table 3. Multivariate Cox analysis of TCRGs after LASSO analysis.

ID	coef	HR	HR.95L	HR.95H	*p*-value
**CTLA4**	−0.407822177	0.665097138	0.524167061	0.843918353	0.000788661
**PDE3B**	0.245124841	1.277780823	0.975172663	1.674292045	0.075462413
**ZFP36**	0.176830915	1.193429285	0.95400983	1.492933733	0.12165874
**BCL11B**	−0.363514764	0.695228463	0.527431354	0.916408575	0.009898316
**SAMD3**	0.472144543	1.603429132	1.011571712	2.541574611	0.044545882
**CMTM3**	0.362024675	1.436234381	1.152730895	1.789462923	0.0012516
